# Glycated hemoglobin and associated risk factors in older adults

**DOI:** 10.1186/1475-2840-11-13

**Published:** 2012-02-06

**Authors:** Raul A Martins, John G Jones, Sean P Cumming, Manuel J Coelho e Silva, Ana M Teixeira, Manuel T Veríssimo

**Affiliations:** 1 Faculty of Sport Sciences and Physical Education, University of Coimbra, Estádio Universitário, Pavilhão 3, 3040-156 Coimbra, Portugal; 2 Department of Biochemistry, University of Coimbra, Coimbra, Portugal; 3 School of Health, University of Bath, Bath, UK; 4 Faculty of Medicine, Hospitals of the University of Coimbra, Coimbra, Portugal

**Keywords:** Glycated hemoglobin, Older adults, Risk factors, Functional fitness

## Abstract

**Background:**

The aim of this study is to investigate the relationships between HbA1c and other risk factors like obesity, functional fitness, lipid profile, and inflammatory status in older adults. Epidemiological evidence suggests that HbA1c is associated with cardiovascular and ischemic heart disease risk. Excess of body weight and obesity are considered to play a central role in the development of these conditions. Age is associated with several risk factors as increased body fat and abdominal fat, deterioration of the lipid profile, diabetes, raising in inflammatory activity, or decreased functional fitness.

**Methods:**

Data were available from 118 participants aged 65-95 years, including 72 women and 46 men. Anthropometric variables were taken, as was functional fitness, blood pressure and heart rate. Blood samples were collected after 12 h fasting, and HbA1c, hs-CRP, TG, TC, HDL-C, LDL-C, and glycaemia were calculated. Bivariate and partial correlations were performed to explore associations amongst the variables of interest. Differences between groups were explored by performing factorial analysis of variance.

**Results:**

HbA1c levels ranged from 4.6%-9.4% with 93% of the cases below 6.5%. Women had higher HbA1c, glycaemia, TC, BMI, and lower and upper flexibility than men. Men had higher BW, WC, 6-min walking distance, and VO2peak than women. Age, SBP, DBP, HRrest, HRpeak, HDL-C, LDL-C, TG, TG/HDL-C ratio, Log10 hs-CRP, upper and lower strength, and agility and dynamic balance were similar in men and women. HbA1c had positive associations with glycaemia, HDL-C, TG/HDL-C, BW, WC, BMI, but not with functional fitness, TC, LDL-C, Log10 hs-CRP, PAD, or PAS. Obese participants had higher HbA1c than non-obese only when IDF and not USDHHS criteria were applied.

**Conclusions:**

Older women had higher HbA1c than men, even after controlling for BMI. HbA1c associates equally with BW, BMI or WC. Population-based criteria are recommended to classify obesity and to identify higher levels of HbA1c in obese older adults. HbA1c associates with atherogenic dyslipidemia particularly with TG and TG/HDL-C ratio, but not with TC, HDL-C, or LDL-C. HbA1c is not associated with hs-CRP, and with functional fitness and aerobic endurance.

## Background

Fasting plasma glucose (FPG) and the oral glucose tolerance test (OGTT) are considered to be appropriate tests for diagnosing pre-diabetes and/or diabetes while OGTT is also considered an appropriate test for assessing diabetes risk in patients with impaired fasting glucose (IFG) [1]. As an alternative to these methods, an International Expert Committee, including representatives of the American Diabetes Association (ADA), the International Diabetes Federation (IDF), and the European Association for the Study of Diabetes (EASD), recently recommended evaluating glycosylated hemoglobin (HbA1c), with a cut-off point of ≥ 6.5% to diagnose diabetes [2] (the HbA1c of young, lean and healthy subjects is approximately 5.0% [3]). This strategy was endorsed and adopted by the ADA in 2010 [4].

Epidemiological evidence suggests that elevated HbA1c is associated with cardiovascular and ischemic heart disease risk [5]. Both obesity and physical inactivity are considered to play important roles in the prevention and treatment of diabetes, with the ADA [6] recommending that people with HbA1c of 5.7-6.4% undergo moderate weight loss (7% of initial body mass), as well as increasing physical activity to at least 150 min/week of moderate activity.

Ageing is another factor that contributes to variance in HbA1c and diabetes risk. Even in nondiabetic adults with normal fasting glucose, HbA1c steadily increase with age, such that at 70+ years of age it is 5.5% [4], almost attaining the ADA criterion for prediabetes. It should be noted, however, that ageing is also associated with a number of risk factors common to the sedentary/obese lifestyle that are expected to be associated with elevated HbA1c levels, including increased body and abdominal fat [7,8], a more atherogenic lipid profile [9,10], diabetes [11], elevated inflammatory markers [12], decreased cardiorespiratory fitness [13] and reduced physical activity [14,15]. Any, or all, of these risk factors are expected to be associated with elevated HbA1c levels. There is, however, little information as to what extent factors such as obesity or physical inactivity in older adults modify HbA1c levels above and beyond the effect of aging, *per se*. This knowledge is essential for determining whether or not 1) lowering HbA1c by diet and exercise is a realistic goal for obese and inactive elderly subjects and 2), if this is indeed achievable, what should be the target levels HbA1c to attain?

The United States Department of Health and Human Services (USDHHS) established cut-off points of > 88 cm waist circumference for women, and > 102 cm waist circumference for men, combined with a body mass index (BMI) of ≥ 30 kg/m2 to define obesity [16]. Similarly, the International Diabetes Federation (IDF) [17] have recommended specific population-based cut-off points for waist circumference, with suggested values of ≥ 80 cm for European women and ≥ 94 cm for European men. Recognizing these criteria to define obesity, the aim of this study is to investigate, in older women and men, the relationships between HbA1c and other risk factors like obesity, functional fitness, lipid profile, and inflammatory status. A secondary aim of this investigation is to compare HbA1c in obese and non-obese older adults, using different cut-off points for obesity.

## Methods

### Participants

Data were available from 118 participants aged 65-95 years, including 72 women (mean age = 77.5 ± 8.4 years-old) and 46 men (mean age = 75.5 ± 6.8 years-old). Participants were required to provide their written informed consent after being informed about potential risks and/or discomforts associated with their participation. All the participants were from the same institution (St. House of Charity) and were provided with similar diets, in terms of caloric intake and nutrients, controlled by a nutritionist. Participants who were taking medications including aspirin and statins maintained unaltered posology for the period of the study. The study conforms to the laws of the country in which it took place, and was approved by an ethical review board at University of Coimbra. Prevalence of obesity and HbA1c levels were compared for three categories of cut-off points: i) waist circumference > 88 cm in women and > 102 cm in men [16]; ii) BMI ≥ 30 kg/m2 [16]; iii) waist circumference ≥ 80 cm in women and 94 cm in men [17].

### Exclusion criteria

All physical or psychological conditions that may have disallowed ability to perform the requested tests, and medications know to influence functional performance or interpretation of the results were considered exclusion criteria. Participants with impaired glucose tolerance and/or diabetes were also excluded.

### Anthropometry

Anthropometric measurements were performed in a separate room, to ensure the participants' privacy. Body weight (BW) was determined using a portable scale (Seca^®^, model 770, Germany) with a precision of 0.1 kg. Waist circumference (WC) was measured at the narrowest part of the torso, above the umbilicus and below the xiphoid process, using a retractable glass fiber tape measure (Hoechstmass-Rollfix^®^, Germany) with a precision of 0.1 cm. Stature was determined using a portable stadiometer (Seca Bodymeter^®^, model 208, Germany) with a precision of 0.1 cm.

### Functional fitness

Functional fitness was evaluated by the Senior Fitness Test battery described in a previous manuscript [18], and constituted the following tests: i) chair stand test, to assess lower-body strength; ii) arm curl test, to assess upper body strength; iii) chair sit-and-reach test, to assess lower-body flexibility; iv) back scratch test, to assess upper-body flexibility; v) 8-ft up-and-go test, to assess agility and dynamic balance; vi) 6-min walk test, to assess the aerobic endurance. Peak oxygen uptake (VO2peak) was estimated with the equation developed by Cahalin and colleagues [19].

### Blood pressure and heart rate

The auscultation method was used to assess resting systolic (SBP), diastolic (DBP) blood pressure, and resting heart rate (HRrest) by using sphygmomanometer (Aneroid Sphygmomanometer-HICO HM 1001^®^, Germany) and stethoscope (Nurse Type Professional Stethoscope-HICO HM-3005^®^, Germany). Participants were in a seated position, consistent with the American College of Sports Medicine (ACSM) recommended procedures for the assessment of resting blood pressure and heart rate [20]. Peak heart rate (HRpeak) was assessed by telemetry with Polar^® ^monitors (Polar S-810i, Finland).

### Blood sampling

Measures were performed as described previously [21]. Venous blood samples were collected into EDTA containing tubes, in the morning between 8:00 am and 9:30 am, after 12 h fasting, and after a minimum of 48 h since the last physical exercise intervention. Participants were in a seated position and rested for five minutes. The cholesterol assessments were direct enzymatic clearance tests conducted at the Randox Laboratories. Total cholesterol (TC) was determined using a Trider-based (CHOD-PAP) colorimetric end-point assay (CH 3810, Randox Laboratories Ltd, UK). High-density lipoprotein cholesterol (HDL-C) was determined using a direct two-point kinetic assay kit (CH 2652, Randox Laboratories Ltd, UK). Low-density lipoprotein cholesterol (LDL-C) was determined with a direct two-point kinetic assay kit (CH 9702, Randox Laboratories Ltd, UK). Triglycerides (TG) were determined using a Trinder-based (GPO-PAP) colorimetric end point assay (TR 3823, Randox Laboratories Ltd, UK). High-sensitivity C-reactive protein (hs-CRP) was assessed via immunoturbidimetry using a high-sensitivity CRP kit (Randox Laboratories Ltd, UK). HbA1c was determined by immunoassay method (HA 3830, Randox Laboratories Ltd, UK). All the methods were controlled and validated using external controls from INSA and RIQAS.

### Statistical analysis

Variables were tested for normality and homogeneity. Variables with distribution significantly different from the norm were log transformed, which occurred with hs-CRP (Log10 hs-CRP). Data are presented as mean values with standard deviations and statistical significance was set at the *p *≤ 0.05 level. Comparisons between females and males were performed with a factorial MANOVA, adjusted by age and BMI on HbA1c, glycemia, hs-CRP, and lipid profile. Comparisons between obese and non-obese participants, with the three categories of cut-off points of obesity (IDF on waist, USDHHS on waist, and USDHHS on BMI), were conducted with factorial ANCOVA, controlling for age and sex. Similar procedures were adopted to compare participants across different levels of aerobic fitness. Associations between HbA1c and other risk factors for cardiovascular disease were calculated with bivariate correlation, and with partial correlation adjusted by age and sex. Data analysis was performed using SPSS v19.0 (SPSS inc, Chicago, IL, USA).

## Results

Baseline characteristics of the participants are summarized in Table 1. Men had higher BW, WC, 6-min walking distance, and VO2peak values than women. In contrast, women had higher BMI, lower and upper flexibility, TC, glycemia, and HbA1c values than men. The age, SBP, DBP, HRrest, HRpeak, HDL-C, LDL-C, TG, TG/HDL-C ratio, Log10 hs-CRP, upper and lower strength, and agility and dynamic balance values were similar in men and women.

**Table 1:** Participant characteristics

	Women (n = 72)	Men (n = 46)	p-value
Age (yrs)	77 (8)	75 (7)	0.36
BW (kg)	69.6 (11.2)	76.7 (9.0)	0.00**
BMI (kg/m^2^)	30.2 (4.0)	28.5 (3.4)	0.05*
WC (cm)	91.0 (9.2)	97.0 (6.2)	0.00**
SBP (mmHg)	149 (17)	149 (23)	0.94
DBP (mmHg)	78 (11)	77 (10)	0.67
HRrest	70 (10)	66 (11)	0.07
HRpeak	115 (21)	119 (22)	0.41
HbA1c (%)	5.7 (0.9)	5.3 (0.3)	0.00**
Glycemia (mg/dL)	101 (25)	91 (12)	0.01**
TC (mg/dL)	216 (37)	209 (45)	0.01**
HDL-C (mg/dL)	53 (9)	49 (10)	0.21
LDL-C (mg/dL)	94 (32)	93 (42)	0.06
TG (mg/dL)	126 (77)	113 (50)	0.21
TG/HDL-C	2.57 (2.10)	2.50 (1.40)	0.44
Log10 hs-CRP	0.17 (0.11)	0.21 (0.19)	0.14
6-min distance (m)	354 (97)	406 (106)	0.01**
VO2peak (mL/kg/min)	14.6 (2.9)	16.2 (3.3)	0.01**
Upper strength (reps/30s)	15 (4)	15 (4)	0.38
Lower strength (reps/30s)	12 (4)	13 (4)	0.66
Upper flexibility (cm)	-20.4 (10.8)	-29.3 (14.2)	0.00**
Lower flexibility (cm)	-5.5 (12.6)	-17.9 (13.7)	0.00**
Agility and dynamic balance (s)	8.64 (3.43)	8.49 (4.53)	0.85

Data are mean (standard deviation)

Differences between women and men: ** *p *≤ 0.01; * *p *≤ 0.05

HbA1c levels ranged from 4.6% to 9.4% with a skewed distribution, with a mean of 5.6%, and 93% of the cases below 6.5%. Considering the wide range of age of the participants (65-95 years), partial correlations were performed controlling for the effect of the sex and age. HbA1c had positive associations with glycemia (r = 0.80; *p *= 0.00), TG (r = 0.26; *p *= 0.02), HDL-C (r = -0.31; *p *= 0.01), TG/HDL-C (r = 0.29; *p *= 0.01), BW (r = 0.34; *p *= 0.00), WC (r = 0.33; *p *= 0.00), and BMI (r = 0.31; *p *= 0.01). HbA1c did not correlate significantly with any component of functional fitness: upper strength (r = 0.09; *p *= 0.45), lower strength (r = 0.08; *p *= 0.51), upper flexibility (r = -0.13; *p *= 0.86), lower flexibility (r = -0.02; *p *= 0.89), agility and dynamic balance (r = 0.07; *p *= 0.54), VO2peak (r = 0.05; *p *= 0.68). HbA1c also did not associate significantly with TC (r = 0.07; *p *= 0.52), LDL-C (r = 0.12; *p *= 0.28), Log10 hs-CRP (r = 0.10; *p *= 0.39), PAS (r = 0.40; *p *= 0.73), and PAD (r = 0.04; *p *= 0.70). Finally, HbA1c did not correlate significantly with age (r = -0.08; *p *= 0.46).

**Figure 1:**
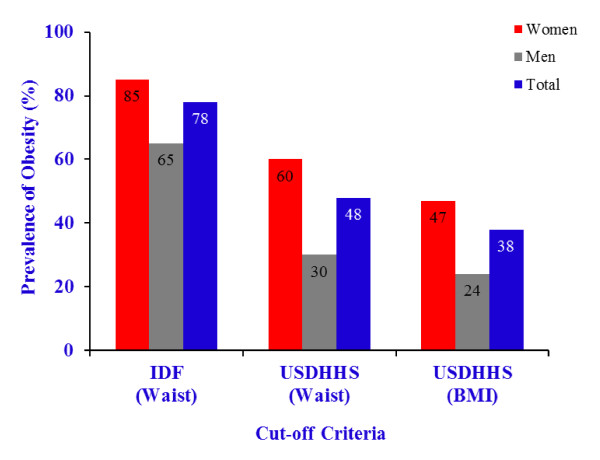
**Prevalence of obesity with IDF **[17]**criteria for central obesity (waist circumference of ≥ 80 cm in women, and of ≥ 94 cm in men), and USDHHS **[16]**criteria (waist circumference of > 88 cm in women men > 102 cm; and BMI ≥ 30 kg/m^2^)**.

**Figure 2:**
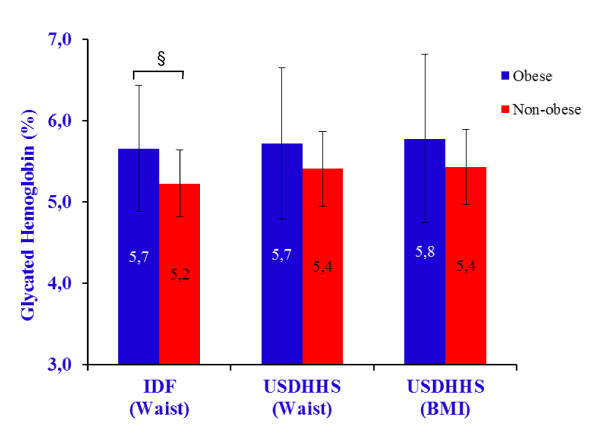
**Glycated hemoglobin by obesity with cut-off points of IDF **[17]**on waist circumference and of USDHHS **[16]**on waist circumference and BMI**. § *p *≤ 0.05.

The prevalence of obesity, using the IDF [17] cut-off points based on WC for European people, was 78% (85% in women and 65% in men), decreasing to 48% (60% in women and 30% in men), and 38% (47% in women and 24% in men) with the USDHHS criteria [16], using WC and BMI, respectively (Figure 1). HbA1c was compared between obese and non-obese people using the three separate categories of cut-off points (Figure 2). Differences between obese and non-obese participants on HbA1c levels, adjusting for age and sex, were only observed with IDF criteria for waist circumference (5.7 ± 0.8% *vs *5.2 ± 0.4%) (F = 3.90, *p *= 0.05), and not with USDHHS criteria for waist circumference (5.7 ± 0.9% *vs *5.4 ± 0.5%) (F = 1.82, *p *= 0.18), nor BMI (5.8 ± 1.0% *vs *5.4 ± 0.5%) (F = 2.21, *p *= 0.14). The IDF and USDHHS criteria for obesity did not interact with sex and age.

**Figure 3:**
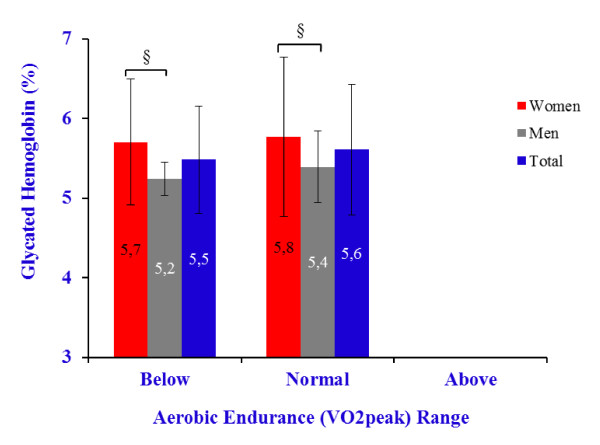
**Glycated hemoglobin by aerobic endurance range **[22]. § *p *≤ 0.05.

Cardiorespiratory fitness is often defined in terms of the VO2peak. The majority of the participants (65%) presented mean values of VO2peak that were 'below average' (68% of women and 60% of men), whereas a smaller proportion (35%) were within 'normal range' (32% of women and 40% of men). None of the participants had 'above average' scores for their respective age group [22]. Figure 3 illustrates HbA1c levels according to the VO2peak level. HbA1c had similar values in older adults below average (5.5% ± 0.7%) and in those within normal range (5.6% ± 0.8%) on VO2peak (F = 0.99; *p *= 0.32). Given the observed small-to-moderate inverse relationship between VO2peak and BMI (r = -0.18; *p *= 0.06), the levels of VO2peak were compared again controlling for the BMI. However, once again HbA1c was similar in both below average and within normal range of VO2peak (F = 0.59; *p *= 0.45).

## Discussion

The results of the current investigation demonstrated that amongst older adults, no sex differences were observed for age, SBP, DBP, HRrest, HRpeak, HDL-C, LDL-C, TG, TG/HDL-C ratio, Log10 hs-CRP, and strength. Males typically present higher mean values of 6-min walk distance, VO2peak, BW and WC, yet lower BMI values, than females. Contrarily, females presented higher values of HbA1c, glycaemia, TC, and flexibility. The results of the current study also suggest that HbA1c is not affected by the age, once the relationship between HbA1c and age was not significant. These results are quite surprising once some have found similar HbA1c in older women and men [23,24], besides a significant increase with age [4,23,25], while others have referred lower HbA1c levels associated with female sex [26]. Consistent with the observation of Kim and colleagues [25], the females in the current study continued to present higher a mean value for HbA1c than males, even when BMI and age were controlled (F = 4.87, *p *= 0.03). However, it is important to note that referred studies focused on people with diabetes, with HbA1c ≥ 6.5%, which is quite different of the present participants.

An examination of the correlational analysis revealed that moderate and positive relation between WC and HbA1c was equivalent in size and magnitude to the correlation observed between HbA1c and traditional predictors of HbA1c risk (i.e., BW & BMI). This observation suggests that regional distribution of fat mass may be an equally valid predictor of HbA1c risk in older adults, and no more or less relevant to HbA1c than BW or BMI. In support of these contentions, other studies have reported similar findings in adults ≥ 60 years, where diabetes is commonly associated with higher BMI and/or WC values [15].

Atherogenic dyslipidemia characterized by elevated TG and low HDL-C has been associated with insulin resistance [27], even with low LDL-C, and may provide clinically relevant information related to the cardiovascular risk. There is literature associating poor HbA1c levels with atherogenic dyslipidemia, specifically with the TG/HDL-C ratio [28]. Other studies have found associations with cardiovascular disease in patients with hypercholesterolemia, suggesting that the control of HbA1c, independently of lipid management, is necessary in order to reduce the cardiovascular risk particularly in diabetic patients with elevated HbA1c [29]. Accordingly, we have described significant associations of the HbA1c with TG, and TG/HDL-C ratio, but not with TC, HDL-C, or LDL-C, which seems to confirm the importance of screening for atherogenic dyslipidemia. The presence of the HbA1c in the model of diabetes assessment could help identify participants at high risk, with the predictability being improved by inclusion of lipid profile [30]. Therefore, evaluating the relationship between HbA1c and lipid profile might be expected to help in the identification of people at cardiovascular risk. That said, our results seem to confirm this importance only to the TG and TG/HDL-C ratio, and not to the other parameters.

Obese participants had higher HbA1c levels than non-obese only when using the IDF cut-off points [17] for waist circumference in European people. However, when using the criteria advanced by the USDHHS [16] (based on waist circumference or on BMI), the obese and non-obese participants did not differ on HbA1c levels, at a significant level. This observation supports the need of using population-specific cut-off points. Clearly, participants comprised between the USDHHS cut-off [16] and the IDF cut-off [17] are influencing the differences on HbA1c levels between obese and non-obese people, which could addresses for wrong conclusions. With respect to the BMI criteria to analyze differences on HbA1c, maybe the value of 30 kg/m^2 ^is not the most accurate taking in account the higher variability usually accompany the older adults. One would speculate that using lower BMI cut-off value would produce differences on HbA1c between obese and non-obese people.

Age has been associated with deterioration in cardiorespiratory fitness [13]. Time spent in sedentary activities has also been reported as increasing with age [31,32] and as a predictor of obesity, atherosclerosis, and cardiovascular disease [33,34]. Some studies have found that weakening of the cardiovascular system associated with aging could be countered by increasing levels of physical activity and functional fitness [21,35,36]. About two thirds (65%) of the participants in this study were below the average on cardiorespiratory fitness, while the remaining 35% within normal range, according to Rikli and Jones [22] cut-off points. HbA1c did not relate with any component of the functional fitness, including flexibility, strength, agility and dynamic balance, and VO2peak, even after controlling for age and sex. Gao and colleagues [5], studying a population with HbA1c mean of 5.8% and 91% of values below 7%, have partially agreed concluding that HbA1c was not associated with risk of developing instrumental activities of daily living (IADL) and/or activities of daily living (ADL) impairment in the whole sample population, however it was associated when analyzing only women. Poor glycemic control (HbA1c ≥ 8%) has been found to explain approximately 10% of disability, i.e. difficulty to perform a physical task by participants aged ≥ 60 years, increasing to 85% when including comorbidities, mostly cardiovascular disease and obesity [15]. In the present study, however, the absence of association between HbA1c and functional fitness, and particularly with cardiorespiratory fitness, could be explained, at least in part, by the HbA1c levels clearly below than those referred above. Additionally, it remains the need to further understand the effect of sex on the relationship between functional fitness and HBA1c in older people.

Low-grade chronic inflammation has been associated with diabetes type 2, with hs-CRP playing a central role increasing acute phase response [37]. Some authors have documented the relationship between hs-CRP and diabetes type 2, with or without obesity [38,39]. More recently, increased levels of hs-CRP in people with increased HbA1c have also been observed [40]. Contrary to expectations, in the present study HbA1c levels were not associated with hs-CRP. However, once again, it would be important to note that participants in this study had lower baseline values of hs-CRP and HbA1c, which could explain this differences.

This study presents two main limitations. It is a cross-sectional one, and in addition, its population is small and includes over 60% of women. Despite such limitations, we believe these data are representative of the prevalence of the reported data in an adult elder cohort.

## Conclusions

The present study suggests that women had higher HbA1c than men, even after controlling for BMI. HbA1c associates equally with BW, BMI, or WC. HbA1c associates also with atherogenic dyslipidemia, particularly TG and TG/HDL-C ratio, but not with TC, HDL-C, or LDL-C. HbA1c is not associated with hs-CRP, or with functional fitness. Obese participants had higher HbA1c than non-obese only when IDF and not USDHHS criteria were applied, highlighting for the importance to use population-specific cut-off points.

## Competing interests

The authors declare that they have no competing interests.

## Authors' contributions

RAM participated in the design of the study, participated in the exercise protocols, performed the statistical analysis and drafted the manuscript. MTV, MJCS and AMT participated in the design of the study and helped to draft the manuscript. JGJ and SPC participated in the draft of the manuscript and revising it critically for important intellectual content. All authors approved the final manuscript.
